# Obsessive Compulsive Treatment Efficacy Trial (OCTET) comparing the clinical and cost effectiveness of self-managed therapies: study protocol for a randomised controlled trial

**DOI:** 10.1186/1745-6215-15-278

**Published:** 2014-07-10

**Authors:** Judith Gellatly, Peter Bower, Dean McMillan, Christopher Roberts, Sarah Byford, Penny Bee, Simon Gilbody, Catherine Arundel, Gillian Hardy, Michael Barkham, Shirley Reynolds, Lina Gega, Patricia Mottram, Nicola Lidbetter, Rebecca Pedley, Emily Peckham, Janice Connell, Jo Molle, Neil O’Leary, Karina Lovell

**Affiliations:** 1 School of Nursing, Midwifery & Social Work, The University of Manchester, Manchester, UK; 2 Centre for Primary Care, The University of Manchester, Manchester, UK; 3 Department of Health Sciences, University of York, York, UK; 4 School of Epidemiology and Health Sciences, The University of Manchester, Manchester, UK; 5 Department of Psychology, University of Sheffield, Sheffield, UK; 6 School of Psychology and Clinical Language Sciences, University of Reading, Reading, UK; 7 Insight Healthcare, Newcastle Upon Tyne, UK; 8 Cheshire and Wirral Partnership NHS Foundation Trust, Wallasey, UK; 9 Anxiety UK, Manchester, UK; 10 ScHARR, University of Sheffield, Sheffield, UK; 11 Norwich Medical School, University of East Anglia, Norwich, UK

**Keywords:** Obsessive compulsive disorder, Guided self-help, Computerised cognitive behaviour therapy, Cognitive behaviour therapy, Exposure and response prevention

## Abstract

**Background:**

UK National Institute of Health and Clinical Excellence guidelines for obsessive compulsive disorder (OCD) specify recommendations for the treatment and management of OCD using a stepped care approach. Steps three to six of this model recommend treatment options for people with OCD that range from low-intensity guided self-help (GSH) to more intensive psychological and pharmacological interventions. Cognitive behavioural therapy (CBT), including exposure and response prevention, is the recommended psychological treatment. However, whilst there is some preliminary evidence that self-managed therapy packages for OCD can be effective, a more robust evidence base of their clinical and cost effectiveness and acceptability is required.

**Methods/Design:**

Our proposed study will test two different self-help treatments for OCD: 1) computerised CBT (cCBT) using OCFighter, an internet-delivered OCD treatment package; and 2) GSH using a book. Both treatments will be accompanied by email or telephone support from a mental health professional. We will evaluate the effectiveness, cost and patient and health professional acceptability of the treatments.

**Discussion:**

This study will provide more robust evidence of efficacy, cost effectiveness and acceptability of self-help treatments for OCD. If cCBT and/or GSH prove effective, it will provide additional, more accessible treatment options for people with OCD.

**Trial registration:**

Current Controlled Trials: ISRCTN73535163. Date of registration: 5 April 2011

## Background

Obsessive compulsive disorder (OCD) is a chronic and disabling mental health condition which exhibits a chronic course unless adequately treated [1]. The obsessions and compulsions that characterise this disorder lead to marked distress, are time consuming and significantly interfere with an individual's functioning. OCD affects approximately 1.1 to 3.0% of the population [2,3].

National Institute of Health and Clinical Excellence (NICE) guidelines for OCD [4] recommend the management of OCD using a stepped care approach [5]. Stepped care is viewed as a potential solution to overcoming poor access to traditional treatments by increasing the efficiency of service provision and benefit to patients. Steps 3 to 6 recommend treatment options for people with OCD that range from low-intensity, primary care-led guided self-help (GSH) to more intensive psychological and pharmacological interventions. Cognitive behavioural therapy (CBT) including exposure response prevention (ERP) is the recommended psychological treatment.

Low-intensity interventions (step 2) are defined by the NICE OCD guidelines as less than 10 hours of therapist time and include CBT (including ERP) with self-managed materials and brief individual CBT by telephone. NICE did not recommend computerised CBT (cCBT) as a treatment, as evidence for cCBT is more limited. A systematic review [6] of cCBT for OCD found only four studies, all using the commercially produced software programme OCFighter (previously known as BT Steps; CCBT Limited, Birmingham, UK (http://www.ccbt.co.uk)), which found that OCFighter showed some benefit in reduction of symptoms and improved work and social functioning. Overall, therapist-delivered CBT was more effective than OCFighter. Although there is some preliminary evidence that self-managed therapy packages for OCD can be effective, a more robust evidence base of efficacy, cost effectiveness and acceptability framed within UK National Health Service (NHS) services is required.

Our study aims to determine: 1) the clinical and cost effectiveness of two self-managed CBT interventions (cCBT and GSH) compared to a CBT waiting list in the management of adults with OCD in the short term at 3- and 6-month follow-up; 2) the clinical and cost effectiveness of self-managed therapies plus conventional CBT compared to waiting list plus conventional CBT at 12-month follow-up; and 3) patient compliance and patient and health professional acceptability of the two self-managed therapy packages (cCBT and GSH).

## Methods

### Design

This study is a multi-centre randomised controlled trial to evaluate the clinical and cost effectiveness of two self-help packages compared to waiting list control for CBT. Informed consent will be obtained from each participant; those who are eligible will be individually randomised to one of three arms: 1) a commercially produced cCBT programme (OCFighter); 2) GSH; or 3) a waiting list for conventional therapist-led CBT.

An internal pilot was conducted to explore the validity of recruitment. This phase of the Obsessive Compulsive Treatment Efficacy Trial (OCTET) was conducted over the first nine months of the recruitment phase and was designed to assess three questions relating to the main trial: 1) is it feasible to recruit the numbers required for a fully powered trial in the designated recruitment time available? 2) Do participants remain on a CBT waiting list for a sufficient length of time (that is, at least 3 months) to conduct an evaluation of the short-term clinical and cost effectiveness of self-managed therapies? and 3) Is it feasible to retain the proposed 6-month outcome as the primary assessment for short term clinical effectiveness?

The pilot was successful and provided evidence that recruitment to target was feasible. At the end of the pilot phase it was identified that it was not feasible to use the proposed 6-month follow-up as the primary outcome assessment, as a significant minority of patients had already reached the top of the waiting list before starting or completing the OCTET interventions. A decision to retain the 3-month assessment as the primary outcome assessment was made.

### Randomised controlled trial

A multi-centre fully randomised controlled trial will be conducted to evaluate two self-managed packages (cCBT and GSH) compared to a waiting list prior to therapist-based CBT.

The key objectives of the OCTET trial are to determine: 1) the clinical and cost effectiveness of two self-managed CBT interventions (cCBT and GSH) compared to a CBT waiting list in the management of OCD patients in the short term at 3- and 6-month follow-up; 2) the clinical and cost effectiveness of self-managed therapies plus conventional CBT compared to waiting list plus conventional CBT at 12-month follow-up; and 3) acceptability of the two self-managed therapy packages (cCBT and GSH) among patients and professionals.

We will randomise patients to: 1) cCBT; 2) GSH; or 3) CBT waiting list. The primary outcome will be OCD symptoms as measured by the Yale Brown Obsessive Compulsive Scale (YBOCS) observer-report version [7].

Table 1 details the secondary outcomes and the tools that will be used.

**Table 1:** Secondary outcome measures

**Secondary outcomes**	**Measured using/by**
Self-reported health-related quality of life	The Short Form (36) Health Survey (SF-36) [8]
Self-reported OCD symptoms	YBOCS self-rated [7]
Generic mental health	Clinical Outcomes in Routine Evaluation (CORE-OM) [9]
Depression	Patient Health Questionnaire (PHQ-9) [10]
Anxiety	Generalised Anxiety Disorder 7 (GAD-7) [11]
Functioning	Work and Social Adjustment Scale (WSAS) [12]
Health-related quality of life	EuroQoL (EQ5D) [13]
Employment status	IAPT Employment Status questions A13–A15 [14]
Patient satisfaction	Client Satisfaction Questionnaire (CSQ-8 UK) [15]
Attachment	Relationship Styles Questionnaire (RSQ) [16]
Perceived criticism	Perceived Criticism Scale (PCS) [17]
Expressed emotion	Family Emotional Involvement and Criticism Scale (FEICS) [18]
Patient progression through mental health services/proportion of patients not improved or partially improved and requiring more intensive CBT	‘Pathway Questionnaire’
Patient and therapist acceptability	Qualitative interviews

#### Three-month follow-up

The 3-month follow-up, as established in the pilot, is the agreed primary outcome point. The primary outcome point reflects CBT waiting lists, and as such will enable us to determine an estimate of short-term clinical and cost effectiveness of the two self-managed therapy packages compared to a CBT waiting list control. This design will not require patients within the trial to have restricted access to treatment beyond that already associated with resource limitations at each site.

#### Twelve-month follow-up

At 12 month follow-up we would expect participants randomised to the waiting list to have been provided with access to conventional CBT. Participants randomised to one of the self-managed packages (either cCBT or GSH) are expected to have either remained on the waiting list and been provided with access to conventional CBT or improved sufficiently with the self-managed package that they no longer require conventional CBT.

The 12-month follow-up will enable us to provide a pragmatic demonstration of the clinical and cost effectiveness of self-managed therapy plus or minus conventional CBT versus waiting list followed by conventional CBT in the longer term. We will be able to explore patterns of longer term outcomes: 1) do patients who access self-managed therapies improve in the short term but relapse in the longer term? 2) Does provision of self-managed therapies augment the benefit from conventional CBT? 3) Do patients who access self-managed therapies maintain longer term outcomes and have less need for conventional CBT than those who remain on a waiting list prior to being offered conventional CBT?

These results will provide critical information concerning the longer term role of self-managed therapy packages for OCD compared to usual care with conventional CBT.

### Inclusion/exclusion criteria

#### Inclusion

Our target population will be adults aged 18 years and above who: meet Diagnostic and Statistical Manual of Mental Disorders (DSM) IV criteria for OCD (assessed using Structured Clinical Interview for DSM (SCID)-IV [19]); score 16 or over on the YBOCS [7] (clinical experience and previous studies suggest that only a minority of people are referred for treatment or excluded from trials with a YBOCS less than 16 (for example, 2.3% [20] 0% [21], 14% [22]); are on a waiting list for therapist-led CBT in either primary or secondary mental healthcare settings; have the ability to read English at a level of 11 years and above.

#### Exclusion

We will exclude patients who: are actively suicidal; have organic brain disease; are experiencing psychosis; meet DSM VI criteria for drug or alcohol dependence [23]; are currently receiving a psychological treatment for OCD; have received CBT for OCD in the last 6 months; have literacy or language difficulties to an extent which would preclude them from reading written or web-based materials or conversing with a health professional.

Patients who have been prescribed or changed to an alternative anti-depressant in the 12 weeks prior to assessment will be excluded, though these patients will be offered a further assessment following 12 weeks of stable medication if there are no plans to increase the medication.

### Recruitment

We will screen all waiting lists in primary and secondary care in our clinical sites and invite potentially eligible participants to a telephone eligibility screen. We will also apply for adoption by the Mental Health Research Network and link with the Primary Care Research Network. These networks, part of the National Institute of Health Research (NIHR), are dedicated to support mental health research projects so we can identify ways in which NHS healthcare can be improved and to ensure maximum patient benefit. They will assist with recruitment from clinical sites to ensure that we fulfil our recruitment target.

If a participant meets the eligibility screen the researcher will give further details of the trial, send information leaflets and a consent form by post and offer them a face-to-face appointment (either in the clinical site or their own home) within seven days from the telephone screen. At the face-to-face interview consent and baseline measures will be taken.

We will recruit a minimum of 432 patients (assuming a 15% attrition rate, total n = 368). Recruitment will occur over a 27-month recruitment period (including the 9-month pilot phase). A £5.00 shopping voucher will be given to each participant completing 3, 6 and 12 months follow-up assessments.

### Randomisation

Patients will be randomised into one of the three arms of the trial: 1) cCBT; 2) GSH; or 3) CBT waiting list.

To ensure the removal of selection bias, allocation will be concealed from the researchers completing assessments by randomisation through a central randomisation service by York Clinical Trials Unit. Details of eligible consenting patients will be entered onto a secure web-based system administered by the Clinical Trials Unit, which will then provide the treatment allocation. Allocation involves minimisation on three important factors - OCD severity (moderate/severe) measured by self-rated YBOCS, anti-depressant medication (yes/no) and depression (mild/moderate/severe) measured by PHQ-9 - with the aim of ensuring a balance across treatment arms.

To reduce detection bias, we will aim to blind researchers undertaking outcome assessments to participants’ treatment allocation. To facilitate blinding, we will use the following procedures: 1) ensuring that the outcome assessments conducted by researchers are completed on different days or locations from the clinical areas in which treatment is being conducted; and 2) asking participants to refrain from revealing their treatment allocation at follow-up assessments. Finally we will test blinding by asking researchers to guess the treatment allocation of the patient at each follow-up and to record the number of patients who inadvertently reveal their treatment allocation.

### Sample size

In trials of non-pharmacological interventions it is recommended that variation between care-providers is considered in sample size estimation and statistical analysis [24]. In this trial there could be variation by therapist (mental health practitioners) in the two self-managed therapies. For the comparisons of CBT waiting list with cCBT or GSH we have assumed between-therapist variation in the active treatment corresponding to a partially-nested design. For the comparison of cCBT versus GSH the same set of therapists will “supervise” both interventions so the trial is therefore a cross-design [25]. Sample size is estimated using the methods described by Walwyn and Roberts [25].

We are not aware of directly relevant estimates for intra-cluster correlation coefficient (ICC) between treatments within therapists, which would be required for sample size estimation for comparison (i) self-managed therapy packages (cCBT versus GSH). We are also not aware of estimates for the ICC for therapists, necessary for sample size estimation for comparisons (ii) CBT waiting list versus cCBT and (iii) CBT waiting list versus GSH. We expect the ICC between treatments within therapists to be less than half the ICC between therapists. With a total sample size of 432 clients and 24 therapists (average of six therapists per clinical site) each with a caseload of six cCBT and six GSH clients the trial will have a power greater than 80% to detect a difference of 3 YBOCS points for each comparison provided the ICC for therapists does not exceed 0.06 and the ICC for treatment within therapists does not exceed 0.015. This calculation assumes a 85% follow-up rate to 3 months (87% was achieved within Lovell and colleagues [20]), a 1.67% significance level to maintain a 5% significance level for three pair-wise comparisons, SD for the primary outcome YBOCS at 3 months of 7.3 units, and a correlation between baseline YBOCS and 3 months YBOCS of 0.43 [20]. In the event that the ICC for therapists is less than 0.1 and the ICC for treatment within therapists is less than 0.05 (which would represent unexpectedly large values), the power is still greater than 75%. By virtue of involving a comparatively large number of therapists the trial is therefore robust against larger ICC values for therapists.

### Interventions

#### Experimental group 1: computerised cognitive behavioural therapy

OCFighter is a commercially produced cCBT programme for people with OCD. OCFighter consists of a nine-step CBT approach (focussed on ERP). Participants randomised to OCFighter will be given an access ID and password to log into the system and will be advised to use the programme at least six times over a 12-week period. cCBT will be offered in one of three locations according to patient choice and local availability: 1) the patients’ own home; 2) the home of a friend or family (if they already have a computer and a broadband connection); 3) the CBT department in the clinical site where the patient is on the waiting list (if a computer in a private room operating on a weekly booking system can be provided). We will actively encourage participants to use cCBT in their own home as the first option.

Participants will receive six, 10-minute brief scheduled support sessions, via telephone or face-to-face (depending on patient preference) from a mental health practitioner (total direct clinical input 60 minutes). The support offered will consist of a brief risk assessment, ensuring that they are able to and have accessed OCFighter, review progress and problem solve any difficulties which are impeding progress.

#### Experimental group 2: guided self-help

The GSH group will consist of a self-help book written by the authors KL and LG. Participants will receive weekly guidance from a mental health practitioner for one initial session of 60 minutes (either face-to-face or via telephone dependent on patient preference) followed by up to ten (30-minute) scheduled telephone or face-to-face (dependent on patient preference) sessions over a 12 week period (total direct clinical input 6 hours). The role of the mental health practitioner will be to conduct a semi-structured interview, devise patient-centered goals and explain the structure and content of the book. They will support them to use the CBT (ERP) interventions described in the book, review progress, pre-empt difficulties as they arise and engage in collaborative problem solving as required.

#### Comparator group 3: waiting list for cognitive behavioural therapy

Our control group will be a waiting list for conventional therapist-led CBT (in both primary and secondary care settings).

### Training and supervision of mental health professionals

The mental health professionals supporting GSH will be individuals who have had mental health training but do not have specialist CBT skills.

Training will be provided for the GSH intervention by trial applicants who have experience and expertise in conducting CBT, GSH and cCBT. cCBT training will be provided for OCFighter by CCBT Limited (manufacturers of OCFighter). Training will consist of 2 days for GSH and 1 day for cCBT and will be delivered at the clinical sites. Training will utilise a range of methods including small and large group work and skill practice with specific feedback using fictitious but typical cases of moderate and severe OCD. Training manuals will be written by the trial team and CCBT Limited and provided for both treatment arms.

Supervision will be provided on a two-weekly basis to the mental health professionals by either clinically qualified trial applicants or senior clinicians within a service.

Researchers and Clinical Study Officers involved in recruitment of participants will be provided with a 1-day extensive training session covering trial procedures and completion of eligibility, baseline and follow-up interviews. A proportion of the day will be spent equipping individuals with the necessary skills to complete the clinician-rated YBOCS. Inter-rater reliability among the researchers/Clinical Study Officers will be employed to measure agreement between ratings for the YBOCS. Top-up training will be provided throughout the trial.

### Concurrent process evaluation

Criticism has been aimed at randomised controlled trials that only focus on pre-specified health outcomes. Process evaluation within trials is recommended to examine key issues such as implementation, acceptability and feasibility, which add to the understanding of the randomised controlled trial results. This study will conduct a process evaluation to explore the barriers and facilitators of implementation by examining the extent to which: 1) patients comply with treatment; and 2) patients and health professionals find treatment acceptable.

#### Patient compliance with treatment

A key aspect of any treatment is the extent to which users complete their agreed course of treatment including CBT between-session tasks. Treatment compliance will be examined through recording the number of sessions attended, and use of self-help materials. With cCBT we will collect automated recordings of the frequency and duration of cCBT use, and patient self-reports of time spent between sessions doing CBT-based tasks. In the GSH arm we will collect number, duration and mode of contact (telephone, email or face-to-face) via mental health practitioner records as well as patient diaries of between-session work.

#### Acceptability of treatments to patients and health professionals

Successful implementation of research into NHS practice requires that new interventions are accepted and welcomed by both patients and mental health professionals. Qualitative interviews will be conducted post-intervention with a subgroup of 10% of patients stratified by baseline severity in both active intervention arms of the trial across clinical sites. Patients will be sampled on characteristics including gender, ethnicity and outcome. We expect to conduct approximately 40 to 48 interviews (11 to 12 interviews at each site). We will also conduct exit interviews with participants who leave treatment early. A sample of 15 to 20 health professionals will be interviewed with the aim of exploring potential barriers and facilitators to implementing GSH and cCBT into clinical practice. Participants will be identified from health professionals who are working in our sites but are not treating patients as part of our trial. The Client Satisfaction Questionnaire will be used to compare patient satisfaction across the treatment arms and, with other quantitative data, to identify predictors of satisfaction.

### Economic evaluation

Little evidence exists regarding the service use or costs associated with OCD, or the cost effectiveness of alternative treatment strategies [26-28], with the exception of a small number of cost of illness or costing studies [29-31], productivity loss [32] and caregiver burden [33] estimates. Clinical evaluations of treatment alternatives for OCD that have included a cost or cost-effectiveness component have been severely limited to the cost of the interventions under evaluation, with no data collected on the impact of the treatment options on the use of other health and social services, patient and family costs, or productivity losses [34-36].

The primary perspective of the economic evaluation will be the NHS/Personal Social Services perspective preferred by NICE. Secondary analyses will take a societal perspective. In line with the key objectives of the proposed trial, the following cost-effectiveness analyses will be undertaken: 1) brief CBT (cCBT and GSH) compared to no active intervention (that is, CBT waiting list) in the management of OCD patients in the short-term (3-month follow-up, as determined during the pilot phase described previously); 2) self-managed therapies plus usual care (waiting list plus access to therapist-led full CBT) versus usual care alone at 12-month follow-up.

Data will be collected on the use of all hospital and community health and social services, productivity losses and costs to patients/families. To the best of our knowledge, no OCD-specific tool for measuring use of services and other resources exists. Instead, the Adult Service Use Schedule (AD-SUS), a generic measure developed by author SB and successfully applied to a range of studies of adult mental health services [37-39], was adapted for OCD through review of relevant literature and discussions with the clinical team to ensure it adequately captures resources appropriate to OCD. The adapted version was tested at baseline interviews with participants in the pilot phase to ensure all important resources are captured. Intervention resources will be collected from therapist records to ensure accuracy and avoid unblinding research assessors.

The unit cost of study interventions will be calculated directly using established methods of micro-costing [40]. Calculations will require data on therapist salaries, including appropriate overheads and employers’ own costs (national insurance and superannuation), working time and estimates of the ratio of direct face-to-face to indirect time. The cost of cCBT will require information on the licensing costs, plus data on any additional purchases of equipment required. For all other health and social services, nationally applicable unit costs will be applied [41-43]. Productivity losses will be calculated using the human capital approach, which involves multiplying days off work due to illness by the individual’s salary [44].

### Statistical analysis

#### Statistical analyses and sample size

A full statistical analysis plan for the analysis of primary and secondary outcome measures including any sub-group analyses will be drafted prior to the commencement of the trial. This will be presented to the Trial Steering Committee (TSC) and the Data Monitoring and Ethics Committee (DMEC) and revised prior to the start of outcome data analysis. During the recruitment and follow-up period regular reports will be prepared for the TSC/DMEC on data quality.

The analyses comparing treatments will be conducted applying the principle of intention to treat. The trial involves the comparison of three treatments and has been powered for a Bonferroni correction for three pair-wise comparisons. Conditional on overall significance at a 5% level, three pair-wise comparisons are planned between: (1) self-managed therapy packages (cCBT versus GSH); (2) CBT waiting list and cCBT; and (3) CBT waiting list and GSH. Inferential analysis will be carried out using a Bonferroni corrected significance level of 0.0167.

Analyses will be performed under the assumption of data missing at random. However, high or differential rates of missing data between treatment arms may indicate a departure from this assumption and may lead to bias. Should this arise sensitivity analyses will be carried out using multiple imputation under a missing not at random mechanism.

For the primary outcome and quantitative secondary outcome measure the treatment effects at 3 and 12 months will be estimated separately using a linear mixed model with a random effect for therapist. To improve the efficiency of the analysis the following baseline covariates will be included in the model for the primary outcome: (1) YBOC score at baseline; (2) anti-depressant medication (yes or no); (3) depression (mild, moderate, or severe); (4) gender; (5) age; and (6) duration of OCD (0 to 5 years, 6 to 10 years, or 10 years and over). Similar covariates will be used for other quantitative measures. A logistic mixed regression model will be used to estimate the effect of the interventions on uptake of CBT by 3 months and 12 months adjusting for baseline severity (YBOCS). Participants will not be excluded from outcome analyses due to missing baseline covariate data. Where baseline covariate data cannot be obtained, imputation involving other baseline covariates will be used. Unless multiple imputation is used for the analysis of outcome, missing baseline data will imputed by single imputation following the suggestion of White and Thompson [45].

Differences in mean costs will be analysed using standard parametric *t*-tests with the validity of results confirmed using bias-corrected, non-parametric bootstrapping (repeat re-sampling) [46]. Despite the skewed nature of cost data, this approach is recommended to enable inferences to be made about the arithmetic mean [47]. Tests will be adjusted for baseline cost, baseline YBOC score and variables potentially predictive of outcome including minimisation variables. Cost effectiveness will be explored in terms of incremental cost per quality-adjusted life year, calculated using the EuroQoL EQ-5D measure of health-related quality of life, and using rules of dominance and extended dominance for a three-arm comparison [48]. Non-parametric bootstrapping from the costs and effectiveness data will be used to generate a joint distribution of incremental mean costs and effects for the three arms. This will then be used to calculate the probability that each of the treatments is the optimal choice, subject to a range of possible maximum values (ceiling ratio) that a decision maker might be willing to pay for a unit improvement in outcome. Cost effectiveness acceptability curves are presented by plotting these probabilities for a range of possible values of the ceiling ratio [49]. These curves are a recommended decision-making approach to dealing with the uncertainty that exists around the estimates of expected costs and expected effects associated with the interventions under investigation [50].

#### Analysis of acceptability interviews

Acceptability interviews will be transcribed verbatim and data will be analysed using Framework analysis [51]. An initial coding framework will be developed and transcripts checked against the framework to ensure that there are no significant omissions. Codes in each interview will be examined across individual transcripts as well as across the entire data set and allocated to the framework. Using aspects of the constant comparative method of analysis broader categories using linking codes will be developed across interviews. Data will be interpreted and analysed within the framework to structure patients’ views about each intervention and reasons for leaving the intervention early.

### Ethics

Ethical approval for this randomised controlled trial has been granted by Lancaster Research Ethics Committee 11/NW/0276.

### Patient and public involvement/user engagement

Service user input, involvement and dissemination of the proposed study comes from Nicola Lidbetter (applicant), Chief Executive of Anxiety UK. We have strong links with Anxiety UK and currently have a number of funded studies (including both a NIHR programme grant and an NIHR Health Technology Assessment programme (HTA) grant) and unfunded collaborative studies (a qualitative study of CBT telephone acceptability for anxiety and depression, applicants KL, PB and NL). In addition we will also invite members of a CCBT OCD subgroup of the Patient and Public Involvement in Research Group in Norfolk who have agreed to be part of a consultation group. Our service user representation will also include assisting us in developing the interview schedules for the acceptability interviews. We will also offer service users training in interviewing if they wish to conduct a proportion of the interviews and they will contribute to the analysis of the interviews and assist in the writing of publications. A proportion of NL’s time will be funded through the grant. A consultancy fee will be paid to participating members of the patient and public involvement in the research group.

### The Trial Steering Committee and Data Monitoring and Ethics Committee

Independent supervision of the trial will be conducted by members of the TSC. The TSC will comprise of an independent chair who has expertise in both trials and OCD and two other independent members including a user representative who has had lived experience with OCD and a clinician working with people with OCD. The principal investigator along with the trial manager will attend and the trial statistician will be invited when appropriate.

A DMEC will be established to assess the progress and safety of the study. It will consist of members external to the study team including a statistician, a clinician and an expert in health services trials. A DMEC report template will be devised for reporting purposes and agreed by the DMEC committee prior to the commencement of the study.

### Forecast execution dates

The set-up of the trial commenced in September 2011 and was complete in January 2012. Recruitment of participants from CBT waiting lists started in a number of sites in February 2012 and will be complete in May 2014. The pilot phase ended in October 2012 at which point recruitment to the full trial began. Follow-up started in May 2012, and the final 12-month follow-ups will be conducted in May 2015. Data analysis will commence in May 2015. Training of mental health practitioners was ongoing throughout the trial with the last training taking place in January 2014.

### Protocol changes

Since the trial commenced a number of protocol changes have been made. These are detailed in Table 2.

**Table 2:** Protocol changes made since the trial commenced

**Aspect of trial**	**Changes made**
Inclusion/exclusion criteria	Individuals no longer excluded if they have received CBT for OCD in the last 6 months
	Individuals no longer excluded if they have started or changed dose of anti-depressant medication within the last 12 weeks
Minimisation criteria	Addition of chronicity to minimisation criteria (defined as duration of OCD: 0 to 5 years/6 to 10 years/10 years and over)
	Primary outcome point changed from 6 months to 3 months following pilot evaluation
Outcomes	Removal of SCID-IV, replaced by Mini-international neuropsychiatric interview (M.I.N.I) and Clinical Interview Schedule Revised (CIS-R)
	Inclusion of Client Satisfaction Questionnaire (CSQ-8 UK) at 6-month follow-up
Recruitment	Submission of mail shot letter to address recruitment issues
	Addition of new recruitment strategies (advert for radio, press and social media)
	Asking participants to wait 12 weeks for their full CBT appointment

## Discussion

The proposed trial is being conducted to explore if the interventions (GSH and cCBT), designed to provide more rapid and efficient access to patients with OCD, are effective. Once complete it will be the largest OCD psychological therapy trial worldwide. Some barriers have been identified that may impact upon recruitment and completion of the trial to time.

### Participant identification methods

The core recruitment method adopted is screening existing CBT waiting lists for potentially eligible patients. These lists were expected to be long and thus containing a large number of patients who may be amenable to participation in OCTET while they waited, as participation allowed them to remain on the waiting list. Similar methods (large scale screening patients from registers or other existing lists of potentially eligible patients) have underpinned a number of our successful trials with other mental health populations.

During the initial stages of the trial set-up, the Department of Health wrote to sites, requiring that patients be offered more rapid access to treatment as part of the Improving Access to Psychological Therapies (IAPT) initiative. The effect was that many patients potentially eligible for OCTET were ‘removed’ from the waiting list and offered access to a ‘low-intensity’ intervention. In many cases, these interventions were in line with current good practice, but were not evidence based, as few were OCD-specific or recommended by the NICE guidelines. However, such patients were lost to the OCTET recruitment procedure, significantly reducing the numbers of potentially eligible patients who could be contacted. In addition, the waiting list initiatives resulted in many patients who were already participating in the trial being offered a CBT appointment prior to the primary outcome point.

### Governance issues

Governance delays also led to delays in the commencement of recruitment. On the assumption that governance agreement should be available within 30 days of submission it was estimated that delays across sites would approximate to 10% loss of capacity (or the equivalent of one site in the initial stages of recruitment).

In response to these issues, which may impact upon trial completion, decisions have been made to recruit additional sites and identify new recruitment strategies such as invitation letters being sent to all patients currently waiting for a CBT appointment, not restricted to those who have an indication of OCD in their NHS records.

## Trial status

We are currently in the final stage of recruiting participants.

## Abbreviations

AD-SUS: Adult Service Use Schedule; CBT: cognitive behavioural therapy; cCBT: computerised cognitive behavioural therapy; CORE-OM: Clinical Outcomes in Routine Evaluation Outcome Measure; CSQ: Client Satisfaction Questionnaire; DMEC: Data Monitoring and Ethics Committee; DSM: Diagnostic and Statistical Manual of Mental Disorders; EQ5D: EuroQoL; ERP: exposure and response prevention, FEICS, Family Emotional Involvement and Criticism Scale; GAD-7: Generalised Anxiety Disorder Assessment; GSH: guided self-help; IAPT: Improving Access to Psychological Therapies; ICC: intra-cluster correlation coefficient; NHS: National Health Service; NICE: National Institute of Health and Clinical Excellence; NIHR: National Institute of Health Research; OCD: obsessive compulsive disorder; OCTET: Obsessive Compulsive Treatment Efficacy Trial; PCS: Perceived Criticism Scale; PHQ-9: Patient Health Questionnaire; RSQ: Relationship Styles Questionnaire; SCID: Structured Clinical Interview for DSM Axis 1 Disorders; SF-36: The short form (36) Health Survey; TSC: Trial Steering Committee; WSAS: Work and Social Adjustment Scale; YBOCS: Yale Brown Obsessive Compulsive Scale.

## Competing interests

The authors declare that they have no competing interests.

## Authors’ contributions

JG manages the trial, leads the service user acceptability evaluation and drafted the manuscript. PBo participated in the design of the trial and assisted with drafting the manuscript. DM coordinates one of the trial sites and assisted with drafting the manuscript. CR participated in the design of the study, developed the statistical analysis plan and assisted with drafting the manuscript. SB leads the economic evaluation and assisted with drafting the manuscript. PBe leads one of the trial sites and the health professional acceptability evaluation. SG assisted with the design of the trial and leads one of the trial sites. CA manages the trial and coordinates data collection. GH and MB assisted with the design of the trial and lead one of the trial sites. SR assists with the coordination of one of the trial sites. LG developed the GSH intervention manual, trained health professionals and assisted with the coordination of one of the trial sites. PM provides research governance support. NL provides service user input. RP, EP, JC and JM are lead researchers and coordinate the collection of data and support researchers at their sites. NO assisted with the development of the statistical analysis plan. KL is chief investigator, led the design of the trial, developed the GSH intervention manual, trained health professionals and assisted with the drafting of the manuscript. All authors read and approved the final manuscript.
